# Long-term cross calibration of HJ-1A CCD1 and Terra MODIS reflective solar bands

**DOI:** 10.1038/s41598-021-86619-y

**Published:** 2021-04-01

**Authors:** Li Liu, Tingting Shi, Hailiang Gao, Xuewen Zhang, Qijin Han, Xinkai Hu

**Affiliations:** 1 China Center for REsources Satellite Data and Application (CRESDA), No.5 Feng Xian East Road, Haidian District, Beijing, China; 2 National Resource Center for Chinese Materia Medical, China Academy of Chinese Medical Sciences, No. 16, Nanxiao Street, Dongzhimen, Dongcheng District, Beijing, China; 3 Aerospace Information Research Institute, Chinese Academy of Sciences, Datun Road 20A, Chaoyang District, Beijing, China; 4 College of Surveying and Mapping Engineering, Guilin University of Technology, Guilin, China

**Keywords:** Space physics, Engineering

## Abstract

Since its launch on September 6, 2008, HJ-1A has been in the orbit for 13 years. The CCD1 sensor on the HJ-1A has four reflected solar bands. Since the calibration frequency is limited to the annual site calibration, cross-calibration is an effective method to improve the calibration frequency. In this paper, we use 420 image pairs of HJ-1A CCD1 and Terra MODIS over the Dunhuang test site for gains calculation, where we take MODIS as the reference sensor. The spectral band adjustment factors (SBAFs) for cross-calibration are then calculated to compensate for the spectral mismatch. The cross-calibration results are also validated by the field calibration results. From 2008 to 2019, a total of six campaigns have been cross-calibrated on the same day. The gain difference between the site calibration and cross-calibration is less than 3%. The long-term cross-calibration results further indicate that due to the adjustment of HJ-1A CCD gain state in October 2009, an abrupt change occurred 405 days after launch. After 12 years of on-orbit operation, the attenuation rate has reached 23.51%, 21.89%, 8.11%, and 13.37%, respectively by the end of 2019 based on the cross-calibration results.

## Introduction

Huanjing-1A (HJ-1A) is a new generation of small civil Earth-observation optical remote sensing satellite in China. It belongs to China’s Environment and disaster monitoring and forecasting satellite constellation project. This satellite constellation consists of two optical satellites (HJ-1A and HJ-1B) and a synthetic aperture radar satellite (HJ-1C). This project aims to improve environmental protection and disaster monitoring and forecasting capabilities. HJ-1A was launched from the Jiuquan Satellite Launch Center on September 6, 2008. It carries two types of payload. One is a charge-coupled device (CCD) composed of two similar charge-coupled devices (CCD1, CCD2). The other payload is a hyperspectral imager (HSI). Since its launch, the satellite has undergone 8-month in-orbit testing by China Center for Resources Satellite Data and Application (CRESDA). By sending high-quality images, it has met the requirements beyond the expectations. Nevertheless, once the sensor is in orbit, attenuation is always expected, therefore radiometric calibration is required. Due to the lack of onboard calibrators in HJ-1A, the in-orbit radiometric calibration of CCD is mainly dependent on the filed calibration^1^. The reflectance-based method is used to measure the Dunhuang test site to calibrate the HJ-1A CCD. The calibration coefficients of HJ-1A CCD obtained by site calibration have been released by CRESDA during 2009–2019. Because HJ-1A site calibration is carried out once a year, the calibration frequency does not meet the requirements of sensor radiation characteristic monitoring. Cross-calibration provides higher calibration frequency and lower cost. Compared with the site calibration, cross-calibration is an effective method. The most common calibration method is to use a well-calibrated instrument as a reference sensor and to cross-calibrate other instruments almost simultaneously. In orbit cross-calibration, near-simultaneous images of common targets on the Earth or Moon surface can be used to cross-reference pseudo-invariant features or data from a third instrument. Among these different cross-calibration methods, the simultaneous nadir overpass (SNO) method is the most effective^2^.

An SNO occurs when the nadir points of two satellites cross each other within a few seconds, which occurs in the polar regions (+ 70° to + 80°, and − 70° to − 80° latitude zones) for sun-synchronous polar-orbiting satellites^2^. It is not suitable for cross-calibration between CCD and MODIS, because most SNO areas are in the polar region. Another method to perform cross-calibration on an invariant target. It is also widely used because it allows cross-calibration in a wider range of conditions by expanding the configuration threshold. This method relaxes the requirement of observation angle as well as the imaging time. The image pair of a non-polar region can be obtained to realize the cross-calibration of the sensor. In general, invariant targets are used to monitor instrument degradation after launch, such as NOAA meteorological satellite^3,4^. In situ measurements can be also used as an intermediate reference for cross-calibration. The most widely used invariant targets for cross-calibration are pseudo-invariant calibration sites (PICS) often located in the African desert^5–8^. The PICS composed of sand dunes that have high reflectance and there is no vegetation with low aerosol in this climate. Assuming that the target radiation is stable, the spectral response and observation geometry adjustment factors are required for the PICS. Most of the PICS calibration in Africa and the Sahara Desert do not rely on the measured data on the ground^5^. Cross-calibration of Terra MODIS and Landsat ETM + reflective solar bands are performed based on pseudo-invariant calibration sites such as Libya1, Libya14, Mauritania 1, Mauritania 2, Algeria 3, Algeria 5 are performed. The results show that the slope of the two sensors is between 2.5 and 15% due to the combination of the RSR characteristics and the spectral characteristics of each sensor^5^. After bidirectional reflectance distribution functions (BRDF) and spectral mismatch adjustment, the calibration consistency between the two sensors has been improved^9^.

The Dunhuang experimental site is selected as the pseudo-invariant calibration site in China. The method was applied to the charged-coupled device (CCD) of the China-Brazil Earth Resources Satellite (CBERS02) on August 19, 2004. Simultaneous ground measurement data are also used to validate cross-calibration results^10^. The cross-calibration coefficient of CCD in HJ-1A/B is used to calculate the calibration coefficients as in^11^. Moreover, since the HJ-1A/B satellite was launched in 2008 and H. Gong’s research work has been done in 2010, it was based on only 2 years’ data which can be used for the study, the change of on-orbit performance cannot be revealed effectively^11^.

In this paper, the cross-calibration of Terra MODIS and HJ-1A CCD1 for long-term images from 2009 to 2019 is performed using the PICS method. The uncertainty caused by the mismatch of spectral response was characterized and compensated.

The organization of this paper is as the following. “Sensor and site overview” section briefly introduces the HJ-1A CCD1, the Dunhuang test site, and existing data. “Methodology” section introduces the cross-calibration method. The SBAF is determined by the spectral mismatch between CCD1 and MODIS. “Results” section presents the calibration results of HJ-1A CCD1. In “Discussion” section the validation results between the site calibration and cross-calibration. Furthermore in “Discussion” section uncertainties of cross-calibration are investigated, including the uncertainties due to the SBAF correction, surface reflectance of Dunhuang test site, aerosol optical depth (AOD), the aerosol model, digital numbers (DNs), MODIS calibration, and Radiative Transfer Model (RTM). “Conclusion” section summarizes the paper and provides the research conclusions.

## Sensor and site overview

### Sensors

**Figure 1:**
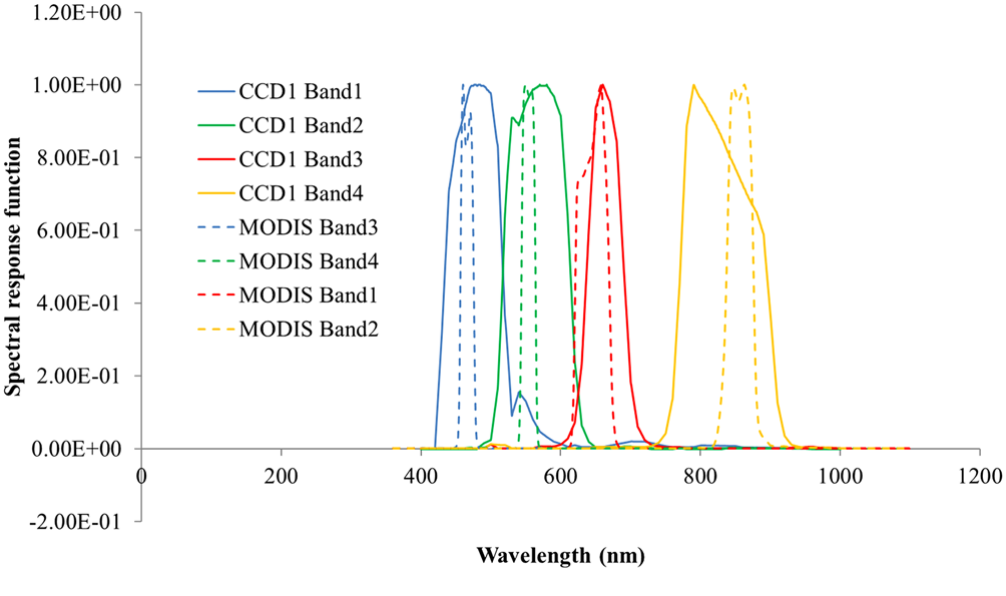
The spectral response function of HJ-1A CCD1 and Terra MODIS.

The CCD sensor onboard the HJ-1A spacecraft has been successfully operated on-orbit for more than 10 years. The HJ-1A CCD is a pushbroom camera that is consolidated by two CCDs (CCD1 and CCD2). Each WFI has 12,000 pixels for linear pushbroom imaging. There are four reflective solar bands, each with 30-m nadir spatial resolution, and ranges from 0.45 to 0.89 μm (see, Fig. 1). The band wavelength centers of the 4 band are 0.49, 0.57, 0.66, and 0.83 μm. The camera has a field of view (FOV) of 32° resulting in a combined swath of 720 km. The revisit period is also less than 4 days. The quantization in CCD is based on 8 bits and the equator crossing time of the HJ-1A spacecraft orbit is 10:30 Local Standard Time (LST).

Cross-calibration involves comparing different observations from a reference instrument, so the choice of reference instrument is essential. Nevertheless, the key feature of the reference instrument is its stability and radiometric uncertainty. Both satellites’ orbits must allow for coverage of the same geographic areas, and the observations should ideally be made at the same time. The relative spectral response (RSR) characteristics should be also considered (see, Fig. 1). MODIS has a robust onboard calibration system and it has an excellent on-orbit performance^12–15^. Note that Aqua MODIS has higher calibration accuracy. However, because Aqua is an afternoon star, it cannot provide a matching calibration time. Therefore, Terra MODIS is selected as the reference instrument for cross-calibration with HJ-1A CCD1, although Terra MODIS has some issues, e.g., solar diffuser door anomaly in May 2003, RVS issues.

Terra MODIS was launched on December 18, 1999, from Vandenberg Air Force Base^16^. MODIS is a cross-track scanning radiometer that has 36 spectral bands with wavelengths ranging from 0.41 to 14.5 μm. The nadir spatial resolutions are 250 m for bands 1–2, 500 m for bands 3–7, and 1000 m for bands 8–36. Bands 1–19 and 26 are the reflective solar bands (RSBs)^17^. The RSB calibration is based on regular measurements from the onboard calibrators (OBs) including a solar diffuser (SD) and a solar diffuser stability monitor (SDSM), and monthly scheduled lunar observations. The calibration uncertainties of MODIS are ± 2% for the TOA reflectance products and ± 5% for the TOA radiance products within ± 45° viewing angles^18^.

Table 1 presents a summary of the key features of both the HJ-1A CCD1 and MODIS sensors.

**Table 1:** Sensor characteristics of HJ-1A CCD1 and MODIS.

Parameter	HJ-1A CCD1		Terra MODIS	
Launched	September 6, 2008		December 18, 1999	
Equator crossing time	10:30 AM (local)		10:30 AM (local)	
Swath	800 km		2330 km	
Altitude	649 km		705 km	
Pixel quantization	8 bits		12 bits	
Bandwidth	Bands	Bandpass (μm)	Bands	Bandpass (μm)
	1	0.43–0.52	3	0.459–0.479
	2	0.52–0.60	4	0.545–0.565
	3	0.63–0.69	1	0.620–0.670
	4	0.76–0.90	2	0.841–0.876
Spatial resolution	Bands	Res (m)	Bands	Res (m)
	1	30	3	500
	2	30	4	500
	3	30	1	250
	4	30	2	250
Exoatmospheric solar spectra irradiance (Esun)	Bands	$Esun_{\lambda }$ (W/(m^2^ µm))	Bands	$Esun_{\lambda }$ (W/(m^2^ µm))
	1	1933.47	3	2087.94
	2	1783.99	4	1865.94
	3	1528.98	1	1606.17
	4	1038.42	2	992.2

We note the following based on Table 1 and Fig. 1:

1. The MODIS swath is significantly wider than that of the CCD1.
2. The MODIS bandpass is significantly narrower than the corresponding CCD1 bandpasses (Fig. 1).
3. Both sensors cross the equator at the same time.

### Dunhuang test site

**Figure 2:**
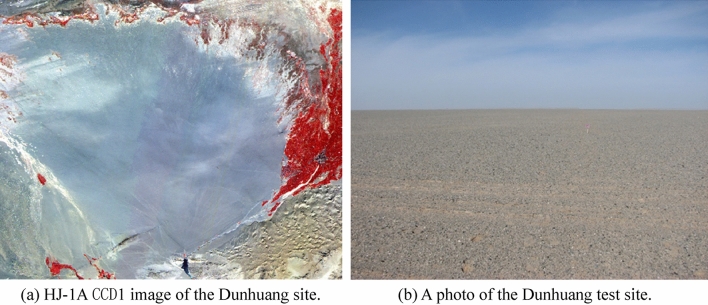
The Dunhuang radiometric calibration field.

The Dunhuang test site is one of the eight instrumented sites from the LANDNET network provisionally selected by the Committee on Earth Observation Satellites (CEOS) (see, Fig. 2). These sites are mainly used for field campaigns. The Dunhuang site (located at 40.04°–40.28° N, 94.17°–94.5° E) is in the west of Dunhuang City in Gansu Province. The site is on an alluvial fan of the Dang River with a uniform area of 25 km^2^ with sparsely distributed vegetation. The study site is in the center of the field area, with an area of 400 m × 400 m and an altitude of 1229 m. The Dunhuang site is constructed of black, gray, and white gravel with a diameter from 0.2 to 8.0 cm^19^. According to the meteorological information acquired at Dunhuang weather station, the atmospheric model ranges from the desert to the continental type. The air is clean and dry and has a lower impact on the atmosphere transmittance.

### Data

The region of interest for cross-calibration is located at 40.092° N, 94.394° E, which is at the center of the Dunhuang test site. Since the spatial resolutions of CCD1 and MODIS are 30 m and 500 m, respectively. A square shape with a 50 pixels side of CCD1 is also selected corresponding to a 3 pixels side of MODIS. The total area is about 2.25 km^2^. The image pairs of CCD1 and MODIS are selected without the constraint for observation geometries to guarantee the availability of enough samples required for the cross-calibration. An outlier filtering algorithm based on a double standard deviation threshold is used to eliminate the abnormal data. The specific method is to calculate the mean value and standard deviation of the normalized gray values of the image. When a certain gray value is greater than the mean value plus 2 times of the standard deviation, or less than the mean value minus 2 times of the standard deviation, the gray value is considered as an abnormal value hence eliminated. The effective gray values are then obtained after removing all abnormal gray values through several cycles. After filtering, the original 700 Gy values are reduced to 420 Gy values. Since the launch of HJ-1A/B in 2008, 420 pairs of CCD1 and MODIS images have been recorded on the ROI of the Dunhuang experimental area. Among the 420 recorded images, 167 CCD1 zenith angles were less than 10 degrees. Furthermore, for MODIS, 51 MODIS zenith angles were less than 10 degrees. If the zenith angle of the sensor is close to 0 degrees, the cross-calibration accuracy will be higher. However, to improve the cross-calibration frequency, CCD1 and MODIS image pairs are selected when the zenith angle of the sensor is less than 10 degrees.

Twelve calibration campaigns are carried out between 2008 and 2019. The in-situ measurement data are used in validating the cross-calibration results which include the ground reflectance, aerosol optical depth, and radiosonde data. The model of the spectroradiometer is SVC HR-1024I. The HR-1024i covers the UV, Visible, and NIR wavelengths from 350 to 2500 nm. It uses 3 diffraction grating spectrometers with 1 silicon and 2 InGaAs diode arrays. The silicon array has 512 discrete detectors and the InGaAs arrays each have 256 discrete detectors that provide the capability to read 1024 spectral bands. The surface reflectance of the test site is the average reflectance collected by transporting the portable spectroradiometer across the entire site over half of a 60-min period during the HJ-1A CCD1’s overpass time. The FOV of the optical fiber is 25°, and the head of the optical fiber is kept at a height of about 0.5 m perpendicular to the ground during measurement.

Sun photometer measurements of the direct solar radiation also provide information to obtain the columnar aerosol optical depth (AOD). The AOD is then used to compute columnar water vapor and estimate the aerosol size using the Angstrom parameter relationship. The CE318 is used in our campaign which is a portable autonomous sun photometer widely used in the AERONET.

Data preprocessing is conducted before the cross-calibration. HJ-1A CCD1 level 1A products and Terra MODIS level 1B products MOD02HKM data set are used for the data processing. The MODIS Level 1B data set contains calibrated and geolocated at-aperture radiances generated from MODIS Level 1A sensor counts. The average DNs are determined from the CCD1 imagery region of interest (ROI) by selecting the Dunhuang test site. Preprocessing of cross-calibration includes three steps: (1) obtaining the gray value and auxiliary information (including imaging date, imaging time, sun and satellite angle information, etc.) of HJ-1A satellite CCD1 sensor; (2) Selecting the satellite image pairs of the CCD and MODIS; (3) obtaining the apparent radiance and auxiliary information (including imaging date, imaging time, sun and satellite angle information, etc.) of MODIS reference satellite.

The gray value of the satellite is directly extracted according to the longitude and latitude information of the image instead of geometric correction. This is due to the following reasons. The first reason is that the Dunhuang site is flat and even, and the area of uniform area is more than 10 km × 10 km. Therefore, carrying out geometric correction has little impact on the final calibration results. The second reason is that geometric correction requires a lot of processing time while does not improve the calibration accuracy. To address these issues, we directly extract the gray value information of the site from the original image. The apparent radiance of MODIS is also obtained based on the calibration coefficients and digital number of MODIS over the Dunhuang test site.

## Methodology

This section introduces the cross-calibration method of the HJ-1A CCD1 and Terra MODIS. The calibration coefficient consists of gain and offset. The offset of HJ-1A CCD1 is determined by laboratory calibration results conducted before the launch. The gain is calculated by cross-calibration and SBAF correction compensation.

### Cross-calibration

Cross-calibration is calculated using spectral radiance or reflectance. Using the TOA reflectance instead of TOA radiance provided the following three advantages^20^. Firstly, it eliminates the cosine effect of different solar zenith angles due to the time difference between the data acquisition cycles. Secondly, the TOA reflectance compensates for the difference of exo-atmospheric solar irradiance caused by the spectral band differences. Third, the TOA reflectance corrects the variation of Earth-Sun distance between different data acquisition periods. These changes are important both geographically and temporally.

The cross-calibration of the HJ-1A CCD1 and Terra MODIS reflective solar bands is carried out and the calibration coefficients are calculated. Assuming that the radiometric response of CCD1 is linear, its calibration formula is expressed as:

1 $${L}_{Ci}=\frac{{DC}_{Ci}}{{g}_{Ci}}+{L}_{0i},$$

where ${L}_{Ci}$ is the TOA radiance of CCD1 i-band; ${DC}_{Ci}$ is the DN of CCD1 i-band; ${g}_{Ci}$ and ${L}_{0i}$ are the TOA radiance gain and the offset of CCD1 i-band, respectively; and i is the band number which is 1, 2, 3, 4.

The TOA radiance is then obtained by the TOA reflectance using the following equation:

2 $${L}_{Ci }=\frac{{\rho }_{Ci }\cdot {E}_{Ci }\cdot \mathrm{cos}\left({\theta }_{C}\right)}{\pi \cdot {d}^{2} },$$

where ${\rho }_{Ci}$ is the TOA reflectance of CCD1 i-band; ${E}_{Ci}$ is the band-specific average exo-atmospheric solar irradiance; d is the average Earth-Sun distance; and ${\theta }_{C}$ is the solar zenith angle.

Modis02 HKM data set further shows that the MODIS reflectance offsets of the CCD1 corresponding band are zero. Therefore, the calibration formula of MODIS normalized TOA reflectance is as follows:

3 $${\rho }_{mi}\cdot \mathrm{cos}({\theta }_{m})={DC}_{mi}\cdot {c}_{mi,}$$

where ${\rho }_{mi}$ is the TOA reflectance of MODIS i-band; ${\theta }_{m}$ is the solar zenith angle of MODIS; ${DC}_{mi}$ is the DN value of MODIS i-band; and ${c}_{mi}$ is the normalized TOA reflectance gain of MODIS i-band.

Suppose that the adjustment factor of CCD1 and MODIS TOA reflectance is k, which represents the spectral mismatch of two sensors, ${k}_{SBAF}$, where ${k}_{SBAF}$ is determined by the TOA reflectance ratio of CCD1 and MODIS that is simulated by a radiative transfer model with the same observation geometry and different spectral response functions. The value of ${\rho }_{Ci}$ is also obtained using^21^:

4 $${\rho }_{Ci }=k\cdot {\rho }_{mi}={k}_{SBAF} \cdot \frac{{DC}_{mi}\cdot {c}_{mi}}{\mathrm{cos}\left({\theta }_{m}\right)}.$$

Combining Eqs. (4), (2), and (1), we then conclude that:

5 $$\frac{k\cdot {DC}_{mi}\cdot {c}_{mi}\cdot {E}_{Ci }\cdot \mathrm{cos}\left({\theta }_{C}\right)}{\pi \cdot {d}^{2}\cdot \mathrm{cos}\left({\theta }_{m}\right)}=\frac{{DC}_{Ci}}{{g}_{Ci}}+{L}_{0i}.$$

There are three key variables in cross-calibration, calibration coefficients ${g}_{Ci}$,${L}_{0i}$, SBAF ${k}_{SBAF}$.

### SBAF correction

The spectral band adjustment factor (SBAF) is the ${k}_{SBAF}$ used for the compensation of the spectral mismatch of two sensors. It is the ratio of the TOA reflectance of the two sensors determined by the radiative transfer calculation^22^:

7 $${k}_{SBAF} =({\rho }_{Ci}^{^{\prime}})/({\rho }_{mi}^{^{\prime}} ).$$

where ${\rho }_{Ci}^{^{\prime}}$, ${\rho }_{mi}^{^{\prime}}$ are the simulated TOA reflectance of the CCD1 and MODIS i-band, respectively.

The TOA reflectance of CCD1 and MODIS is simulated by the 6S radiative transfer model. The input parameters include ground reflectance, atmospheric parameters, and various spectral response function of CCD1 and MODIS^23^. It should be noted that only the view geometry of CCD1 is used to simulate the TOA reflectance of MODIS and CCD1. Among the input parameters for SBAFs calculation, the observation geometries are changed with the changing sensor’s attitude. The atmospheric model also varies according to the month, and it is set to the midlatitude summer from April to September and midlatitude winter from October to March. The SBAFs improves the cross-calibration accuracy between CCD1 and MODIS, which have significantly different relative spectral reflectance (RSR)^24^.

### Validation

The cross-calibration with the SBAF corrections is validated by comparing the results of site calibration and cross-calibration. The validation procedure uses the field calibration results based on the reflectance-based method. Field measurements have been also collected in 12 campaigns since the launch of HJ-1A. However, from 2008 to 2019, there were only six field measurements that occurred on the same day with cross-calibration, namely August 16, 2010, September 13, 2011, August 9, 2014, August 8, 2015, August 16, 2015, July 16, 2018. The results of the site calibration coefficient are then used to validate the cross-calibration.

The reflectance-based method is used to calculate the field calibration coefficients. The average reflectance of each band of HJ-1A CCD1 is calculated by the spectral reflectance curves measured in-situ. The aerosol optical thickness is also measured by solar photometer CE138. The results of these measurements are used as input to the radiative transfer code, for which the output is the sensor radiance prediction value for each band^25^. By selecting 5 × 5 pixels regions at the same time, the average DNs are determined from the region of interest (ROI) of the image. Finally, the sensor gain is calculated by combining the predictions at sensor radiance and DNs.

## Results

This section provides cross-calibration results of CCD1 and MODIS, using SBAF. The SBAF corrections are applied to determine the cross-calibration coefficients on the ROI of the Dunhuang test site by suing the 420 image pairs of the CCD1 and MODIS.

### Data preprocessing

**Figure 3:**
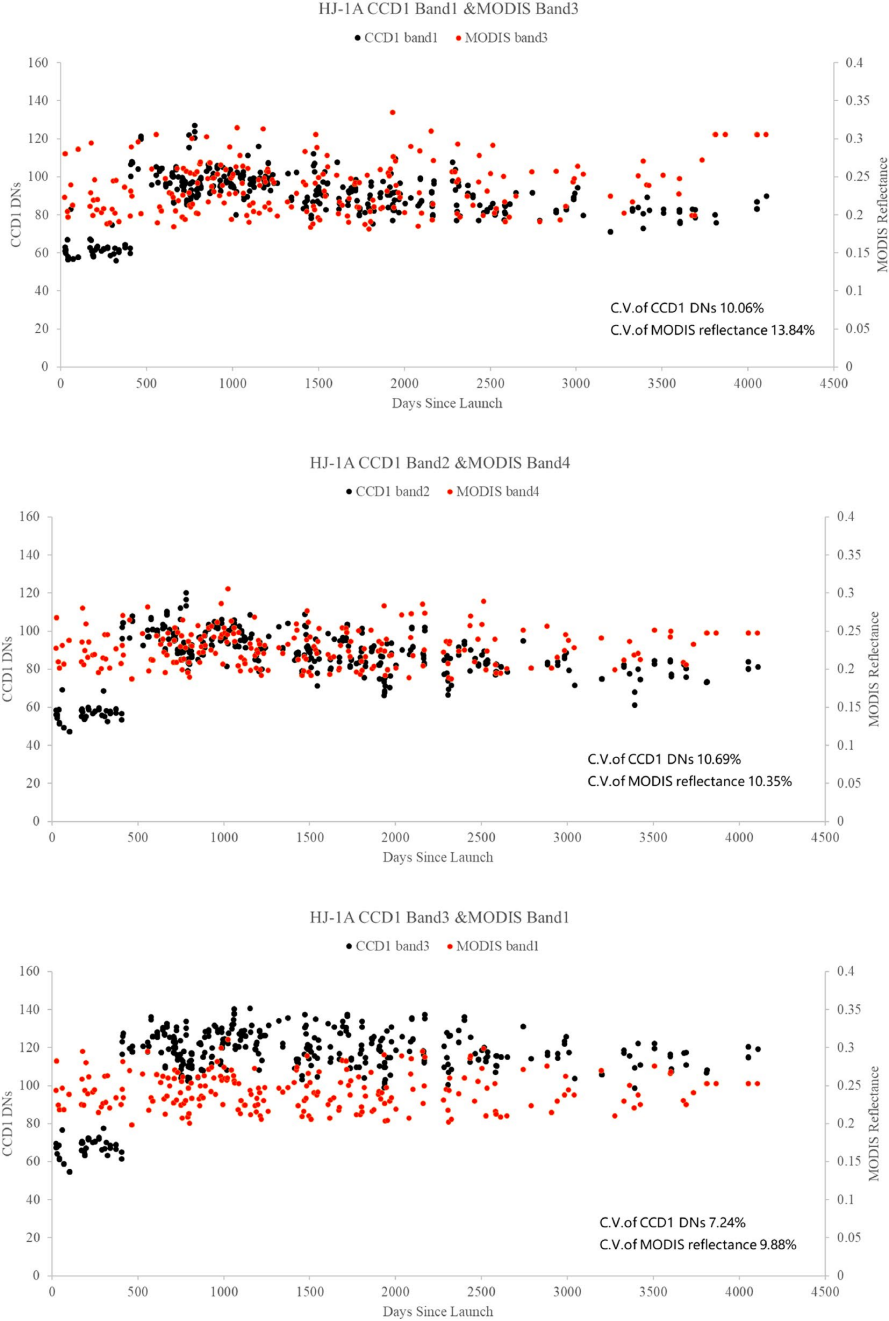

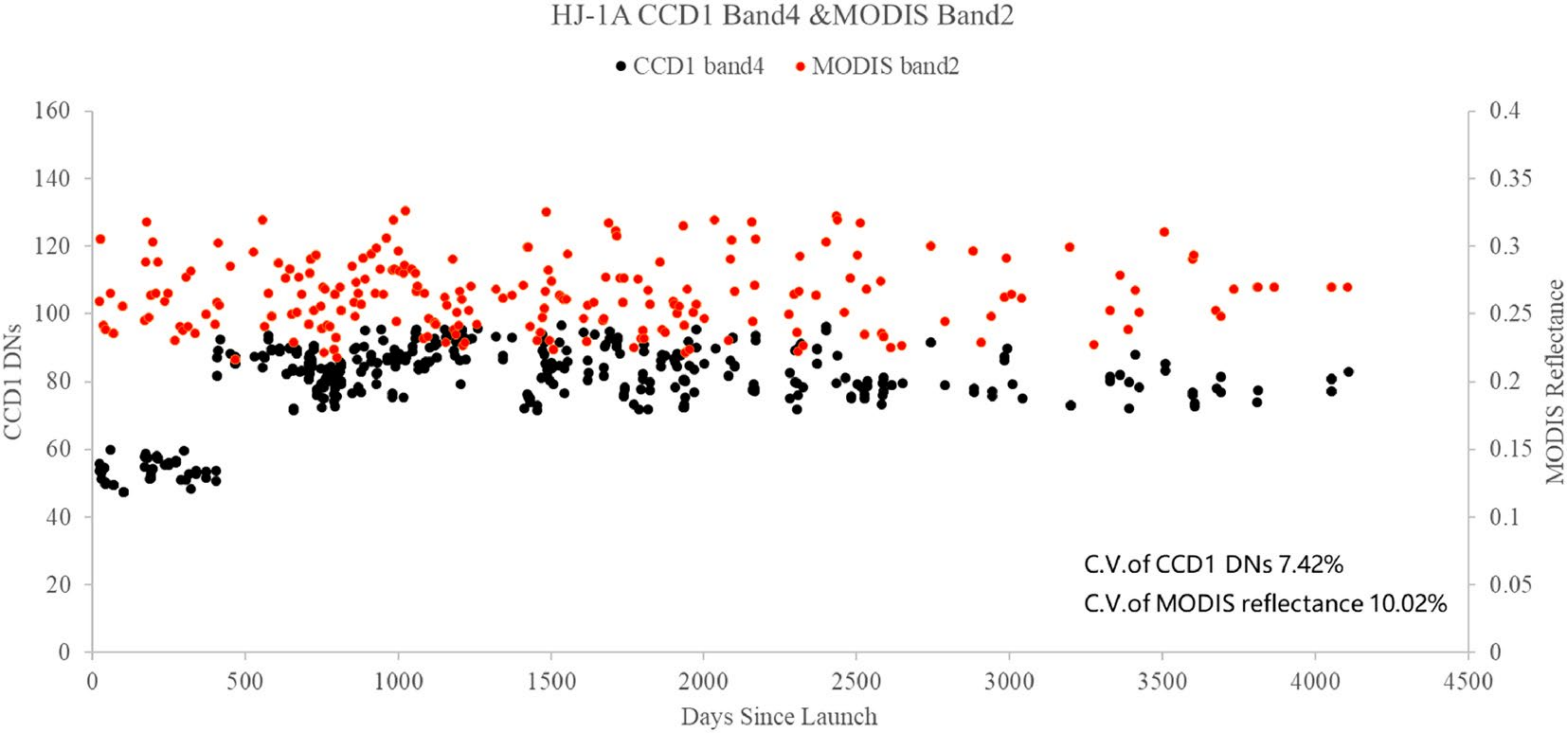
The CCD1 DN and MODIS TOA reflectance determined by the image pairs.

The DN values of ROI are extracted from CCD1 and MODIS images. According to Eq. (3), the TOA reflectance is obtained by MODIS DN and calibration coefficients from the data set of MOD02HKM. The DNs of CCD1 and MODIS TOA reflectance since the launch of HJ-1A are presented in Fig. 3.

The results presented in Fig. 3 indicates the following:

1. The CCD1 DN of the Dunhuang test site shows clear seasonal variations. The DN values of the summer image are higher than those of the winter. Therefore, the normalized DNs are calculated to eliminate seasonal variations. There is also a sharp jump at 405 DSL (day since launch) in the normalized DNs. The changing trend of the DNs after 409 DSL is consistent with that of the MODIS TOA Reflectance.
2. The coefficient of variability (C.V.) is used to analyze the variation of DN. The C.V. of CCD1 for bands 1, 2, 3, 4 are 0.1006, 0.1069, 0.0724, and 0.0742, respectively. The C.V. of MODIS bands 3, 4, 1, 2 are 0.1384, 0.1035, 0.0988, and 0.1002, respectively. The results show that CCD1 DN is more different than MODIS TOA reflectance. However, the C.V. of CCD1 bands 1, 2, 3, 4 had only little differences.

**Figure 4:**
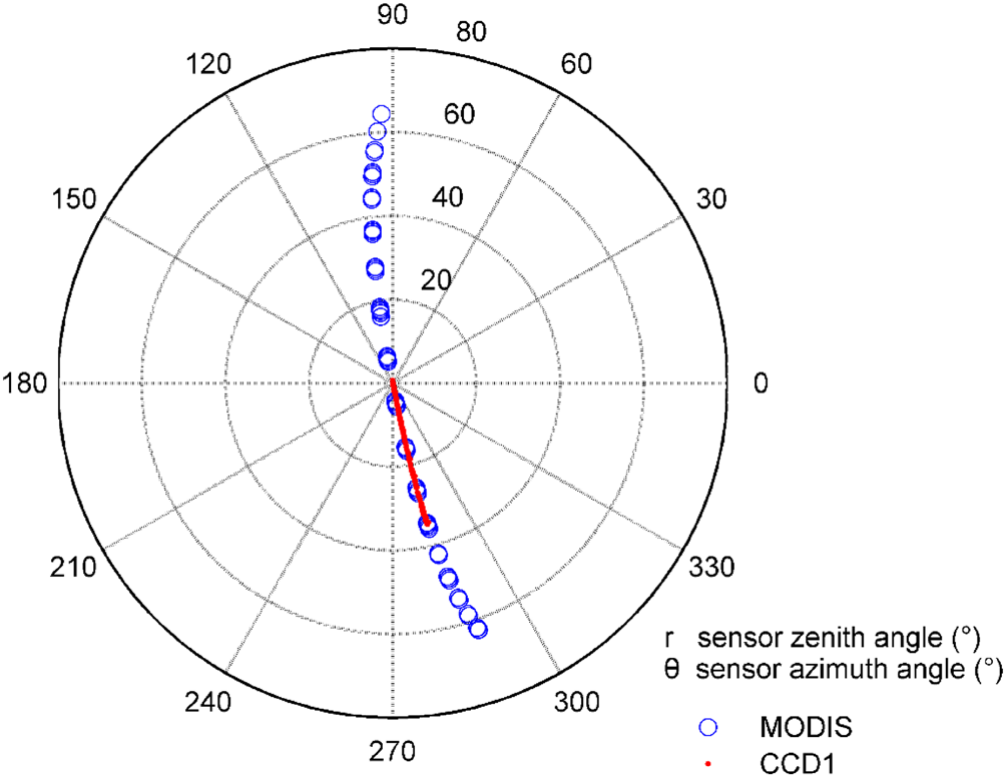
Sensor geometries of CCD1 and MODIS.

**Figure 5:**
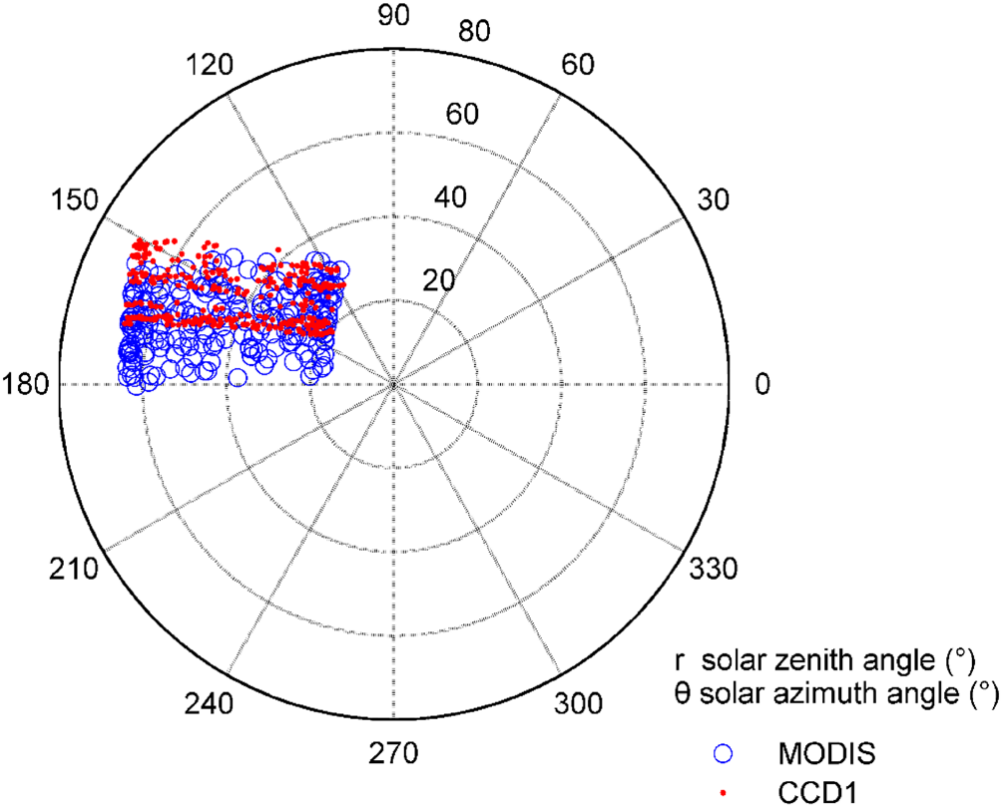
Solar geometries when CCD1 and MODIS overpass.

The observation geometry of CCD1 and MODIS is obtained from the auxiliary files. Taking the azimuth of CCD1 and MODIS as the angle between X-axis and radius vector, and zenith angles as the length of the radius vector, the geometric distribution of sensor geometries is expressed in the polar coordinate system (see, Fig. 4). The geometries of the sun are also seen in Fig. 5.

Figure 4 shows that the zenith angle of CCD1 is less than 35 degrees, while the MODIS zenith angle is less than 65 degrees. Among the 420 collected images, 167 CCD1 zenith angles are within the range of 0–10 degrees, accounting for the largest proportion. Furthermore, 96 CCD1 zenith angles are concentrated in the range of 10–20 degrees and 86 CCD1 zenith angles are concentrated in the range of 20–30 degrees. There are 71 CCD1 zenith angles greater than 30 degrees. At the same time, 51 MODIS zenith angles are less than 10 degrees, and 80 and 57 MODIS zenith angles are concentrated between 10 and 20 degrees and 20–30 degrees. There are 232 MODIS zenith angles greater than 30 degrees, which are accounting for about 50% of the total recorded images. The azimuth angle is mainly concentrated in 90–100 degrees and 280–290 degrees.

Figure 5 shows the solar zenith and azimuth angles of CCD1 and MODIS crossing the Dunhuang test site, and they have similar distribution patterns. In particular, the solar zenith is mainly distributed in the range of 20–70 degrees, and the azimuth is between 110 and 170 degrees. Moreover, the solar geometry of MODIS has a similar distribution. The geometry of the sensor is varied, especially the zenith angles, while the geometry of the sun has a similar distribution.

**Figure 6:**
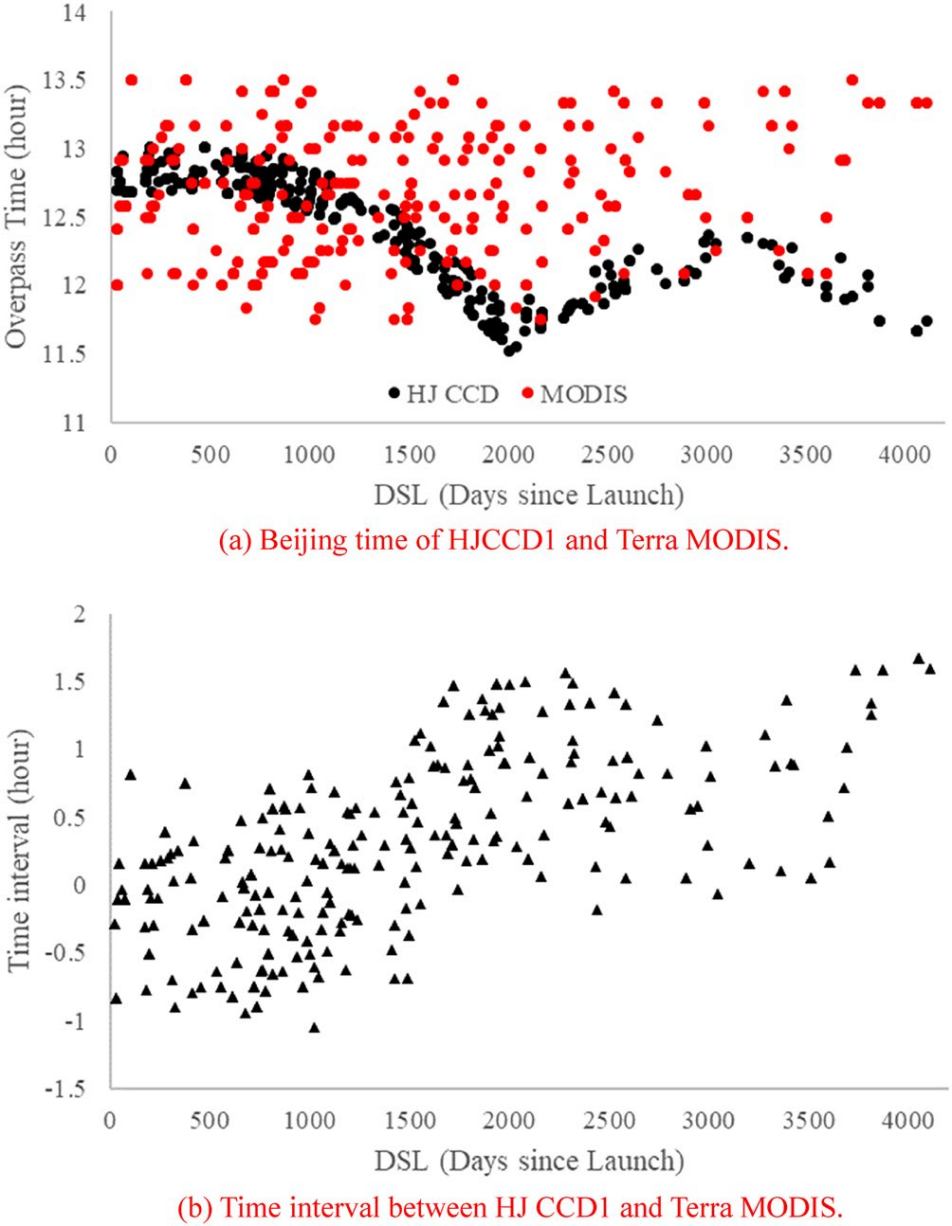
The time difference between the two satellites overpassing Dunhuang.

Figure 6 shows the time difference between HJ1A CCD1 and Terra MODIS overpassing Dunhuang in 420 image pairs. The results show that the imaging time of HJ1A CCD1 images of the adjacent dates changes continuously, whereas the imaging time of Terra MODIS images of adjacent dates changes greatly. The maximum difference of imaging time between HJ1A CCD1 and Terra MODIS is more than 1.5 h. As the cloudless images have been selected in the preprocessing, the assumption that the atmospheric parameters of the Dunhuang site do not change in a period (within 2 h) is considered in this paper. Therefore, during the calculation of the SABF correction coefficient and cross-calibration coefficients, the uncertainty caused by the imaging time difference is ignored.

### Cross-calibration results of SBAF correction

**Figure 7:**
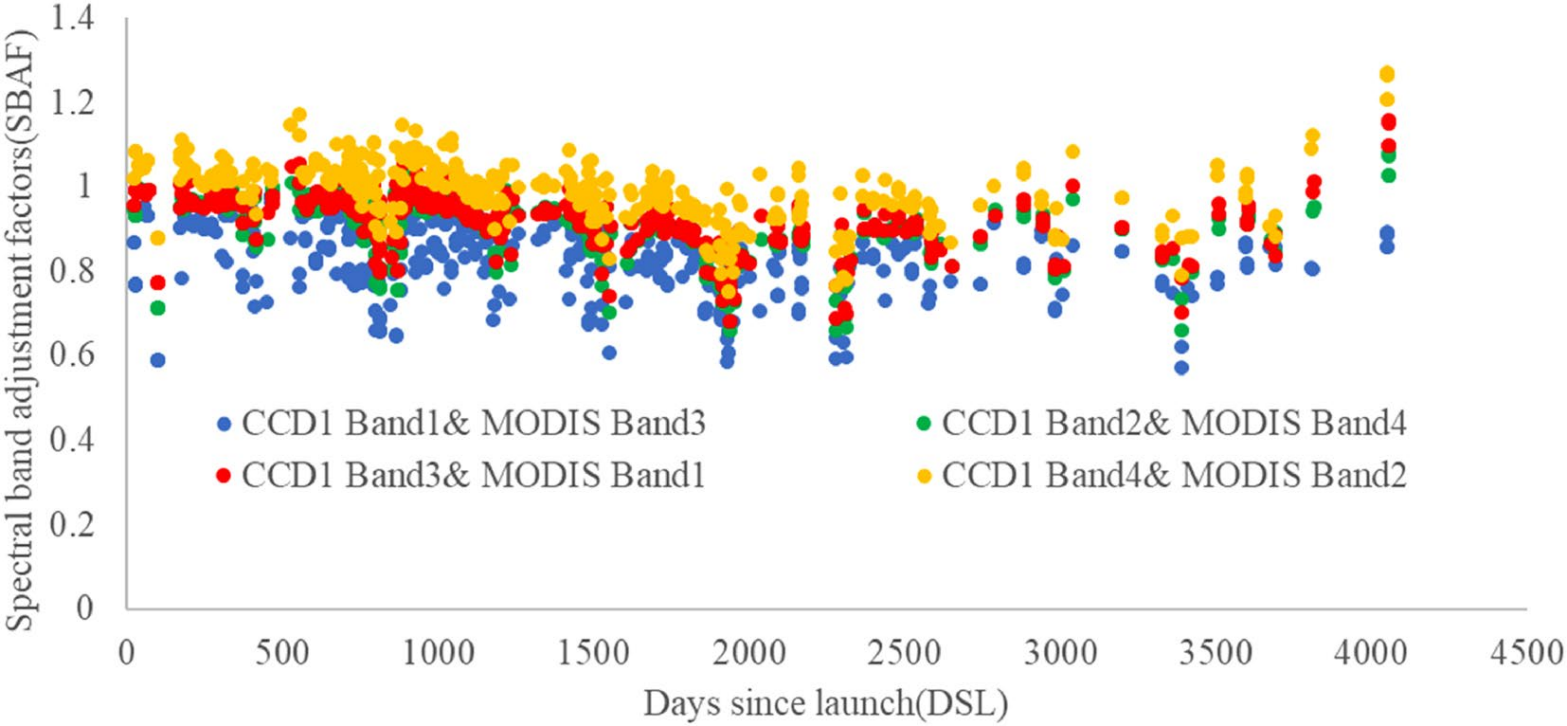
The spectral band adjustment factor of the cross-calibration.

The SBAF correction is used to compensate for the mismatch of the relative spectral response by Eq. (7) (see Fig. 7).

As shown in Fig. 7, the average SBAFs of the four bands are 0.8242, 0.9038, 0.9181, and 0.9866, respectively. The maximum C.V. is 0.1011 which appears at the SBAF of CCD1 band1 and MODIS band3. Most SBAFs C.V. is less than 0.1.

**Figure 8:**
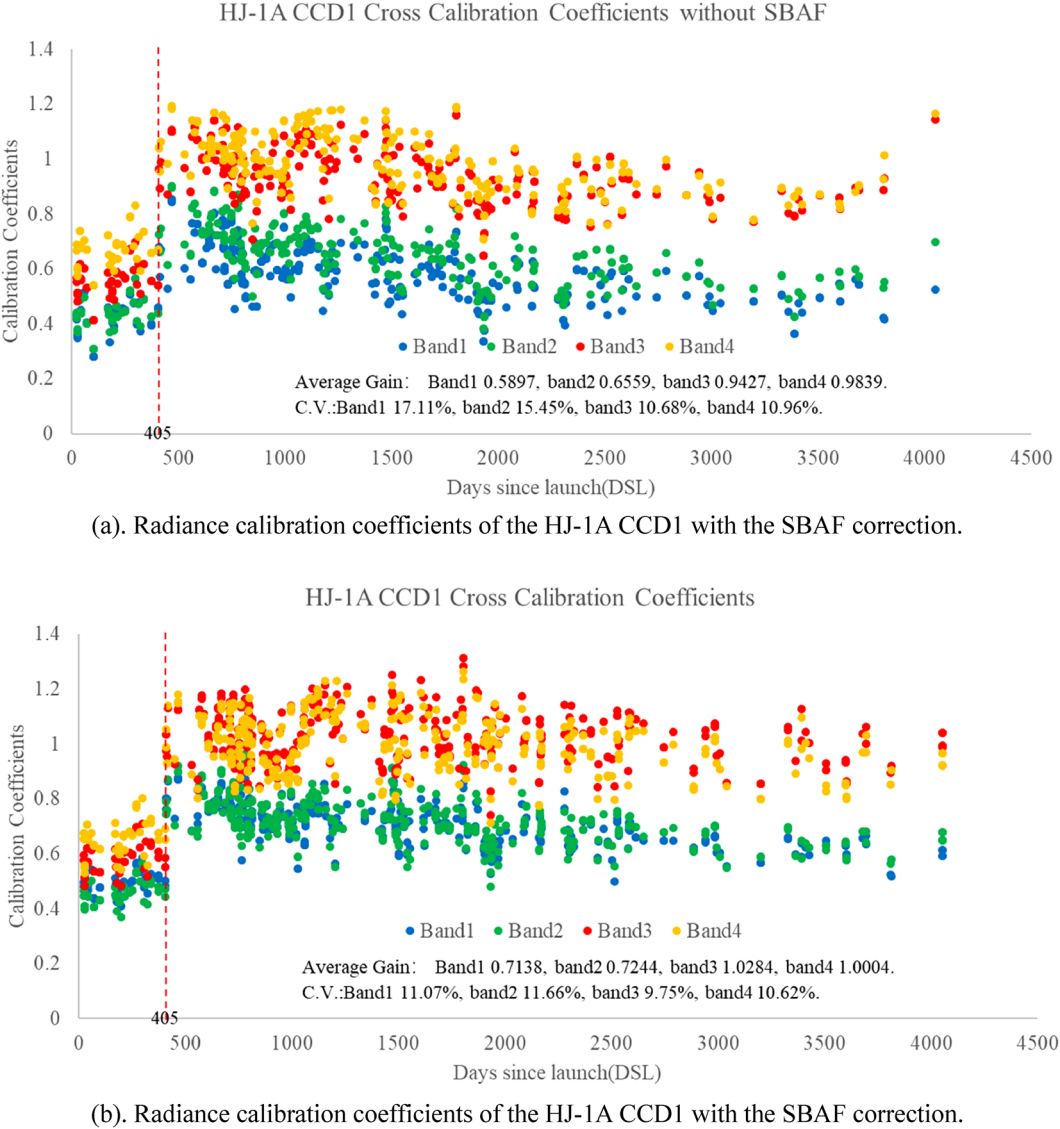
Radiance calibration coefficients of the HJ-1A CCD1 with and without the SBAF correction.

The cross-calibration coefficients of CCD1 and MODIS without and with SBAF correction are shown in Fig. 8.

In 2009, the gain state of HJ-1A CCD was adjusted, and mutation occurred in 405 days after the launch and the amount of the increase was relatively stable. For the HJ-1A CCD1 band1, 2, 3, 4, the average values of calibration coefficients after DSL 405 were 1. 0.7138, 0.7244, 1.0284 and 1.0004 W/(m^2^ sr µm), respectively. The SBAF correction effectively reduces the C.V. of bands 1, 2, 3, and 4 to 11.07%, 11.66%, 9.75%, 10.62%, respectively.

## Discussion

In the previous chapter, the multi-temporal cross-calibration coefficients with SBAF correction are given. In this paper, the cross-calibration results are validated in the field. The uncertainty of cross-calibration is discussed.

### Validation

The sensor radiance is calculated by using the calibration coefficient determined by site calibration and cross-calibration, combined with the average DNs determined by the ROI of the image. At-sensor radiances predicted from the field measurements can be used to validate the cross-calibration results (see Table 2).

**Table 2:** Comparison of at-sensor radiance determined from the site calibration and cross calibration.

Date		Band1	Band2	Band3	Band4
2010/8/16	DN	84.0830	85.0280	103.4720	71.8890
	SC	0.7768	0.7796	1.0312	1.0049
	CC	0.8053	0.8170	1.1065	1.0982
	TOARad_SC	108.24	109.07	100.34	71.54
	TOARad_CC	104.41	104.08	93.51	65.46
	RD	− 3.54%	− 4.57%	− 6.81%	− 8.50%
2011/9/13	DN	75.8056	73.7500	93.7222	62.5556
	SC	0.7696	0.7815	1.0914	1.0281
	CC	0.7575	0.8221	1.1561	1.1377
	TOARad_SC	98.50	94.37	85.87	60.85
	TOARad_CC	100.08	89.71	81.07	54.99
	RD	1.60%	− 4.94%	− 5.59%	− 9.63%
2014/8/9	DN	70.1100	70.8800	96.0000	65.0000
	SC	0.6892	0.7178	1.0072	0.9938
	CC	0.7357	0.7505	1.0809	1.0331
	TOARad_SC	101.73	98.75	95.32	65.40
	TOARad_CC	95.30	94.44	88.81	62.92
	RD	− 6.32%	− 4.36%	− 6.82%	− 3.80%
2015/8/8	DN	69.0000	70.0000	95.6600	64.1600
	SC	0.7050	0.7367	1.0633	1.0436
	CC	0.7199	0.7487	1.1092	1.0224
	TOARad_SC	97.88	95.02	89.97	61.48
	TOARad_CC	95.85	93.49	86.24	62.75
	RD	− 2.07%	− 1.60%	− 4.14%	2.07%
2015/8/16	DN	68.1100	69.3300	96.1100	65.8800
	SC	0.7050	0.7367	1.0633	1.0436
	CC	0.6722	0.6805	1.0035	0.9527
	TOARad_SC	96.61	94.11	90.39	63.13
	TOARad_CC	101.32	101.88	95.77	69.15
	RD	4.87%	8.26%	5.95%	9.54%
2018/7/16	DN	73.4573	75.1645	103.7790	68.5619
	SC	0.6210	0.6070	0.9550	0.9360
	CC	0.6354	0.6425	0.9370	0.8547
	TOARad_SC	118.29	123.83	108.67	73.25
	TOARad_CC	115.60	116.98	110.76	80.21
	RD	− 2.27%	− 5.53%	1.92%	9.50%
	AverageRD	− 1.29%	− 2.13%	− 2.58%	− 0.13%

Rad Unit: W/(m^2^ sr µm).

Note that DN is the digital number of ROI HJ-1A CCD1. The SC and CC are the results of field calibration and cross-calibration. TOARad_SC and TOARad_CC are the top of atmospheric radiance determined by site calibration and cross-calibration. The RD is the relative difference between TOARad_SC and TOARad_CC.

### On-orbit performance of HJ ccd1

**Figure 9:**
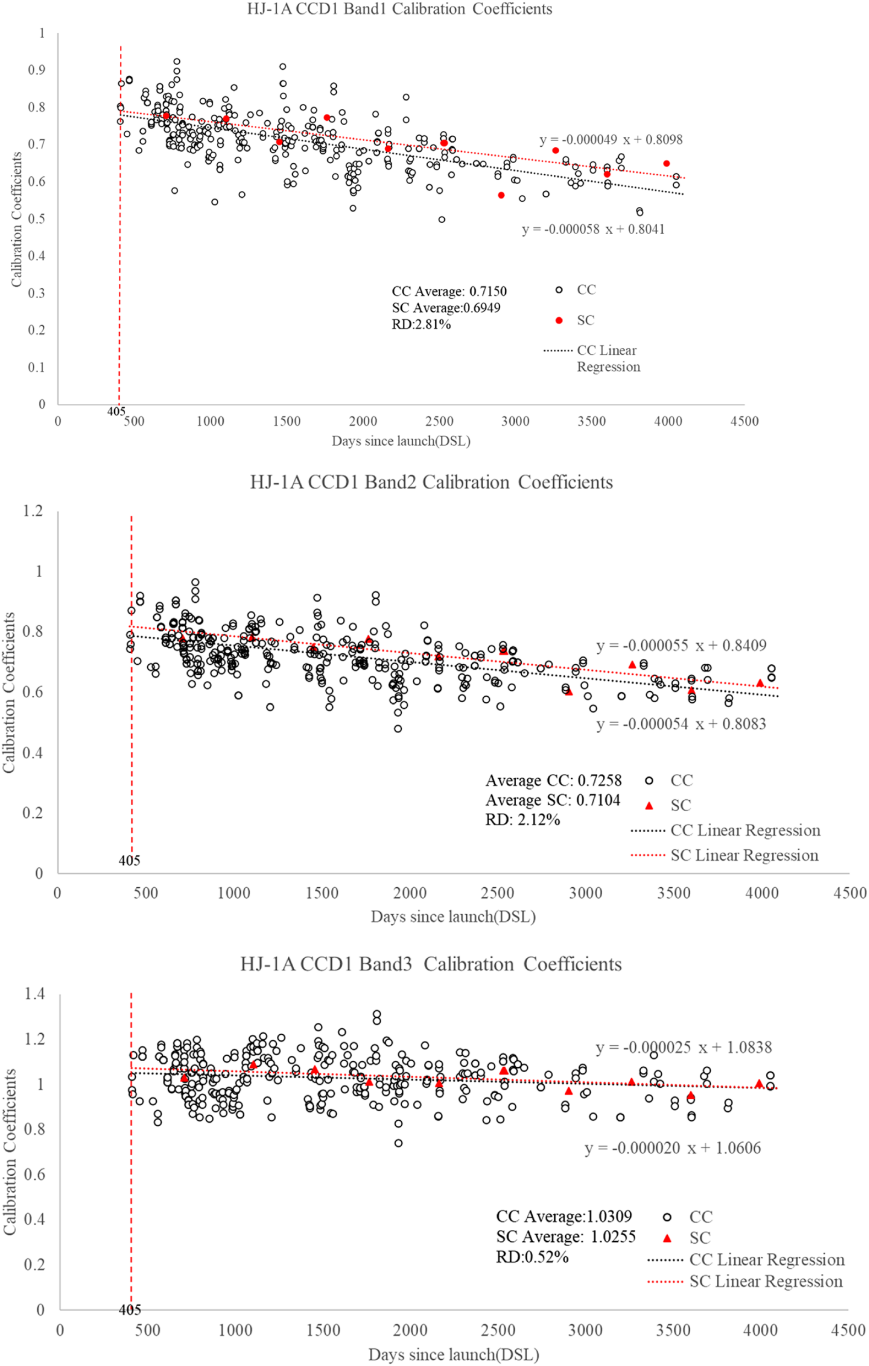

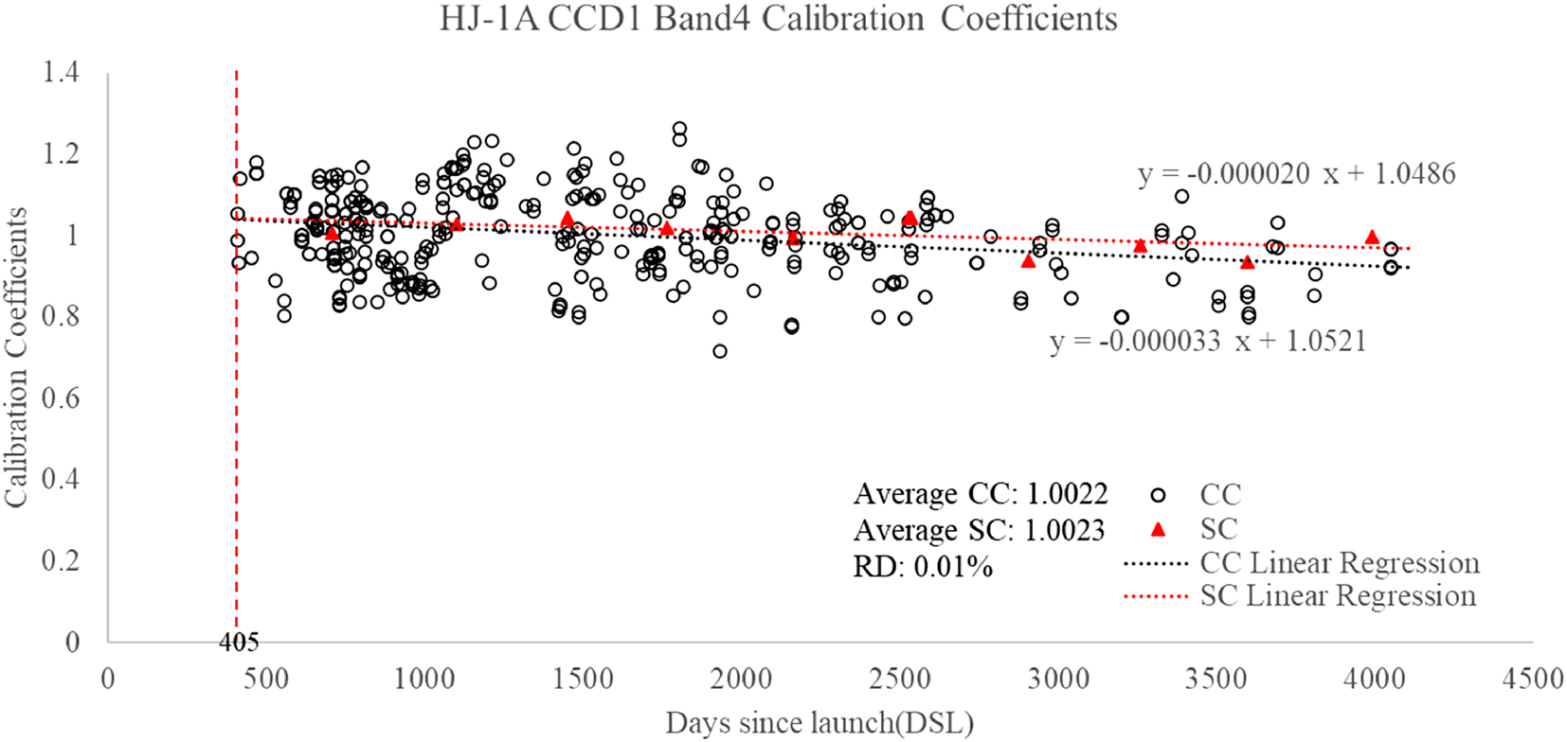
Long-term calibration coefficients for HJ-1A CCD1 bands 1, 2, 3, 4.

HJ-1A CCD1 was launched on September 6, 2008, and has been fully operational since then. During the on-orbit test, the instrument shows good performance. However, the gain state was set to a new mode on October 17th, 2009 to meet the instrument’s imaging requirement. Since then, the instrument has run smoothly. The cross-calibration coefficient was used to analyze the attenuation of the CCD1 (Fig. 9). The trend of the field calibration and cross-calibration coefficient is consistent.

According to the results in Figs. 8 and 9, the daily variance of the cross-calibration results is very high. This is mainly affected by the following factors: (1) the atmospheric change of the site; (2) the influence of the surface directivity; (3) the instability of the undetermined target satellite and reference satellite. Therefore, if only a cross-calibration result for one scene image is used, the uncertainty will be increased. However, after 420 groups of calibration coefficients are linearly fitted, the fitting linear calibration coefficients have good consistency with the site calibration results. Among them, the average calibration results of 420 sets of cross-calibration results of four bands are 0.7150, 0.7258, 1.0309, and 1.0022, respectively. The average values of the four bands were 0.6949, 0.7104, 1.0255, and 1.0023, respectively. The relative differences between them were − 2.89%, − 2.17%, − 0.53%, and 0.01%.

Besides, from Fig. 9, we can also see the time series calibration formula of four bands. The results are shown in Table 3. According to the long-term calibration coefficients of HJ-1A CCD1 bands 1, 2, 3, 4, the linear regression Eq. (8) is determined.

8 $${\text{y }} = {\text{ ax }} + {\text{ B}},$$

where x is the date after the launch of CCD1, y is the estimated calibration coefficient determined by the linear regression equation, a is the daily attenuation, and b is the fitting pre-launch calibration coefficient. The time-series calibration coefficient equation can be found in Table 3.

**Table 3:** Comparison of time series calibration coefficients of HJ-1A CCD1.

Band	Time series calibration equation with cross calibration method	Time series calibration equation with site calibration method
1	Y = − 0.000058x + 0.8041	Y = − 0.000049x + 0.8098
2	Y = − 0.000054x + 0.8083	Y = − 0.000055x + 0.8409
3	Y = − 0.000020x + 1.0606	Y = − 0.000025x + 1.0838
4	Y = − 0.000033x + 1.0521	Y = − 0.000020x + 1.0486

Coefficients Unit: W/(m^2^ sr µm).

According to the time series calibration results based on 420 sets of images, the calculated daily attenuation rates of the four bands are 5.8 × 10^–5^, 5.4 × 10^–5^, 2.0 × 10^–5^, and 3.3 × 10^–5^, respectively. According to the time series calibration results of site calibration, the daily attenuation rates of the four bands are 4.9 × 10^–5^, 5.5 × 10^–5^ and 2.5 × 10^–5^, and 2.0 × 10^–5^, respectively. The above results show that the two methods have a good consistency. The attenuation values of band 1 and band 2 are larger than that of band 3 and band 4. According to Table 3, the pre-launch calibration coefficient of the HJ-1A satellite can also be obtained. Among them, the pre-launch calibration coefficients of the four bands obtained by the cross-calibration method are 0.8041, 0.8083, 1.0606, and 1.0521, while those obtained by site calibration are 0.8098, 0.8409, 1.0838, and 1.0486, respectively. The relative differences in pre-launch calibration coefficients between the two methods are 0.70%, 3.88%, 2.14%, and 0.33%, respectively. This result further proves the reliability of time series calibration results based on the cross-calibration method.

After 12 years of on-orbit operation, the attenuation rate will reach 23.51%, 21.89%, 8.11%, and 13.37% respectively by the end of 2019 based on the cross-calibration equation. When the decay rate reached 19.86%, 22.29%, 10.13%, and 8.11% at the end of 2019 after the 12 years of on-orbit operation, based on the site calibration equation. In the whole lifetime of HJ-1A CCD1, the average values are 0.7150, 0.7258, 1.0309, 1.0022 for cross-calibration and 0.6949, 0.7104, 1.0255, 1.0023 for site-calibration of CCD1 bands 1, 2, 3, 4, respectively. The relative differences between the average cross-calibration and site calibration are 2.81%, 2.12%, 0.52%, and 0.01% for corresponding bands. DSL 409, 2171, 4053 are 3 points near the beginning, middle, and end of the timeline. The cross-calibration coefficients on the 3 DSL points are calculated with SBAF correction. Meanwhile, the cross-calibration coefficients can be estimated by the linear regression Eq. (8). The results are presented in Table 4.

**Table 4:** Comparison of cross-calibration coefficients.

	Band1	Band2	Band3	Band4
CC_DSL409_	0.7835	0.7664	1.0045	1.0202
CCe_DSL409_	0.7804	0.7862	1.0524	1.0521
RD_DSL409_	0.40	− 2.59	− 4.77	− 3.12
CC_DSL2171_	0.6960	0.7103	1.0273	0.9840
CCe_DSL2171_	0.6782	0.6911	1.0172	1.0520
RD_DSL2171_	2.56	2.70	0.98	− 6.91
CC_DSL4053_	0.6029	0.6634	1.0163	0.9433
CCe_DSL4053_	0.5690	0.5894	0.9795	1.0519
RD_DSL4053_	5.62	11.15	3.61	− 11.51

Coefficients Unit: W/(m^2^ sr µm).

## Conclusion

In this paper, the on-orbit cross-calibration of HJ-1A CCD1 and Terra MODIS solar reflective bands since launch was investigated. From September 6, 2008, to October 12, 2019, 420 HJ-1A CCD1 and Terra MODIS images were cross-calibrated. The cross-calibration results were validated by in-situ measurements on August 16, 2010, September 13, 2011, August 9, 2014, August 8, 2015, August 16, 2015, and July 16, 2018. Because these days, the field calibration campaign measure is the same as data as the cross-calibration. The average error of the field calibration and cross-calibration is less than 3%.

Besides, the decay of HJ-1A CCD1 was analyzed using the long-term calibration coefficients. The instrument runs smoothly. However, since the gain state of HJ-1A CCD was adjusted in October 2009, a sudden change occurred 405 days after launch. After 12 years of on-orbit operation, the attenuation rate reaches 23.51%, 21.89%, 8.11%, and 13.37% respectively by the end of 2019 based on the cross-calibration results.
